# A Rapid Review of Environmental Health Gaps in Antimicrobial Resistance and Water-Related Research from 1990–2020

**DOI:** 10.3390/ijerph19116549

**Published:** 2022-05-27

**Authors:** Lina Taing, Himesh Bhatia, Rachel A. Kaiser, Manzoor Qadir, Hamid Mehmood

**Affiliations:** 1 United Nations University Institute for Water, Environment and Health (UNU-INWEH), 204-175 Longwood Rd. S, Hamilton, ON L8P 0A1, Canada; himesh.bhatia@queensu.ca (H.B.); rakaiser42@tntech.edu (R.A.K.); manzoor.qadir@unu.edu (M.Q.); hamid.mehmood@unu.edu (H.M.); 2 School of Geography and Earth Sciences, McMaster University, 1280 Main Street West, Hamilton, ON L8S 4K1, Canada; 3 School of Environmental Studies, College of Interdisciplinary Studies, Tennessee Technological University, 1 William L Jones Drive, Cookeville, TN 38505, USA

**Keywords:** antimicrobial resistance (AMR), One Health, food security, water security, water, sanitation, and hygiene (WASH)

## Abstract

Antimicrobial resistance (AMR) is a pervasive global health threat linked to human antimicrobial misuse and abuse, food production, and broader environmental contamination. While global agencies promote a multi-sectoral One Health system approach to equitably combat human, animal, and environmental health AMR risks, it is widely acknowledged that the human and animal sectors dominate discussions. Given this disproportionate focus, identification of critical research gaps is needed to develop stewardship plans that equitably address One Health AMR threats. This review used natural language processing and term frequency algorithms to classify 12,638 records from 1990–2020 thematically in order to highlight sectoral prioritization and gaps. It also specifically assessed water-related gaps as water is recognized as both a primary environmental dissemination pathway and key means of intervention. Drawing from systemic health and integrated water management lenses, this review found that themes related to plant, wildlife, and environmental-related AMR threats—in particular, the role that environmental (ambient) waters play in AMR development, transmission, and spread—are under-prioritized as compared to human and food animal health concerns regardless of geographic region or income level. Further prioritization of these themes is needed to strengthen the environmental dimension of One Health AMR responses and systemically protect global health.

## 1. Introduction

Antimicrobial resistance (AMR) refers to bacteria, viruses, parasites, and fungi that are resistant to antimicrobial compounds. AMR is a public health crisis that can render medical advancements against diseases such as tuberculosis and malaria ineffective due to increased pathogen resistance [1,2]. While AMR occurs naturally, human antimicrobial overconsumption, misuse, and abuse have accelerated this process [3]. Anthropogenic activities moreover have contributed to the widespread dissemination of AMR organisms and genes across a variety of reservoirs and pathways—from humans, animals, and plants to consumables such as food and water, to places such as surfaces in hospitals and runoff from farms [3]. The growing AMR threat is especially of concern to nascent low- and middle-income countries (LMICs), where rising gross domestic products (GDPs) and healthcare access have contributed to increased and unregulated antibiotic use [4]. At present, an estimated 700,000 people die annually due to infections from drug-resistant microorganisms, which is predicted to increase to 10 million deaths a year and result in an annual GDPs loss of up to $100 trillion by 2050 if increasing AMR risk and dissemination are not effectively curbed [5,6]. 

In response to the emerging AMR crisis, the World Health Organization (WHO), World Organization for Animal Health (OIE), and Food and Agriculture Organization (FAO) have developed a global action plan that espouses a One Health approach to combatting human, animal, and environmental AMR risks in a coordinated multi-sectoral and interdisciplinary manner [7,8,9]. Recent reviews and expert reports, however, have noted that there is limited focus on the role of the environment and natural ecosystems within the current body of literature and policy despite the global One Health push [3,5]. Within the environment sector, water resources, specifically environmental (ambient) source water, are extensively overlooked, preventing an integrated One Health approach [3,4,10]. To ensure that AMR risk is holistically and effectively combatted, there is a need for research and stewardship that addresses all AMR risks—from human to animal to environmental reservoirs and dissemination pathways [3,10,11,12,13]. 

The purpose of this review is to contribute to the broader discussion of One Health AMR stewardship by identifying sectoral priorities and key gaps in human, animal, or environmental health themes in relation to geographic, income, and temporal trends. This review utilized natural langue processing and term frequency algorithms in a rapid review of AMR literature from 1990–2020 to identify thematic gaps in the One Health integrated approach. A deeper dive into water stewardship gaps was also conducted, as water has been identified as the primary environmental AMR reservoir and dissemination pathway but has thus far received limited attention in One Health achievements [3,10]. Specifically, attention was given to the primary role water plays in relation to human and animal health, whether as a source, supply, or as wastewater. The findings from this rapid review can aid the development of multisectoral AMR One Health stewardship plans and integrated management through greater prioritization, resource allocation, and capacity development for under-developed and under-prioritized AMR themes. 

## 2. Methods

### 2.1. Database Rapid Review

**Figure 1:**
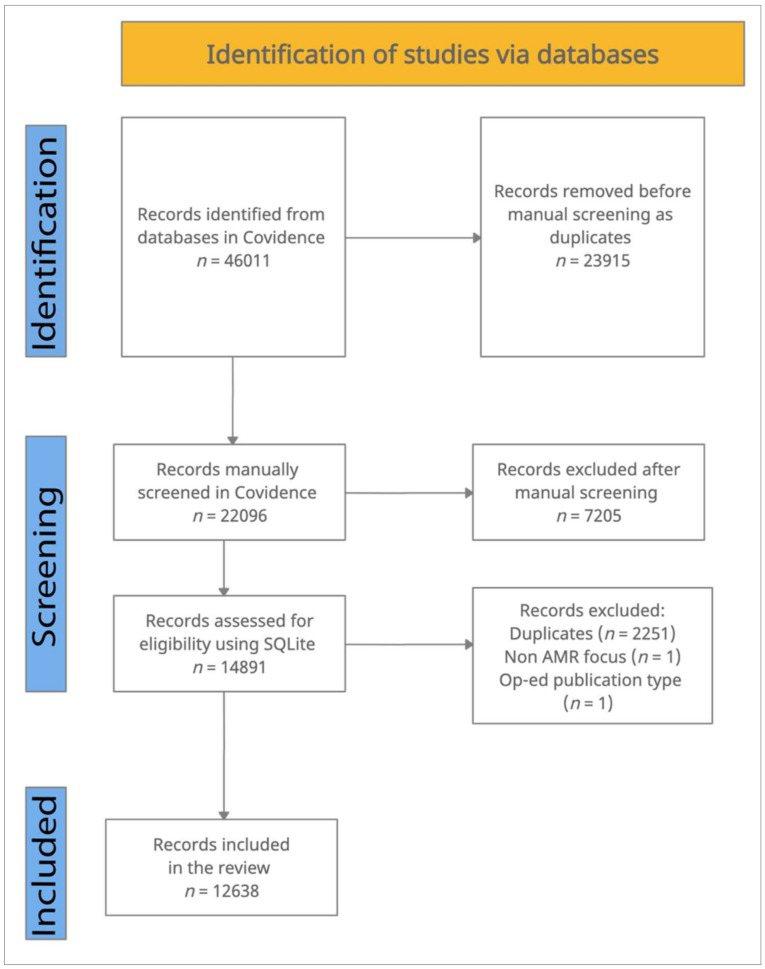
PRISMA diagram outlining the article record selection process.

This study analyzed AMR literature from 1990 to 2020 through a rapid review process [14]. This timeframe was selected because the threat of AMR was acknowledged as a shared global problem beginning in the early 1990s [15]. Titles and abstracts of AMR-focused article records were retrieved from health (Medline, Embase, and Global Health) and interdisciplinary (Web of Science and SCOPUS) databases in September 2020 (Table S1: Database Search Keywords and Search Strings). These article records were manually screened based on criteria related to population, intervention, outcome, data range, and publication type (Table 1) from October 2020 to April 2021 in Covidence. A secondary screening was conducted in SQLite to facilitate synthesis of the large sample and to exclude duplicates based on digital objective identifier (DOI) numbers, limited AMR focus, or publication type. The review included a total of 12,638 article records (Figure 1). 

### 2.2. Overview of the AMR Stewardship Framework Utilized to Guide Data Mining and Article Record Categorization

The review utilized the One Health–One Water AMR Stewardship Framework developed by Kaiser et al. [10] to guide the trend analysis of the included AMR article records. The defined themes (Table 2) [3,5,10,16] and water type definitions [10] (Table 3) were used to categorize the current body of AMR-related literature.

### 2.3. Data Mining and Article Record Categorization

**Figure 2:**
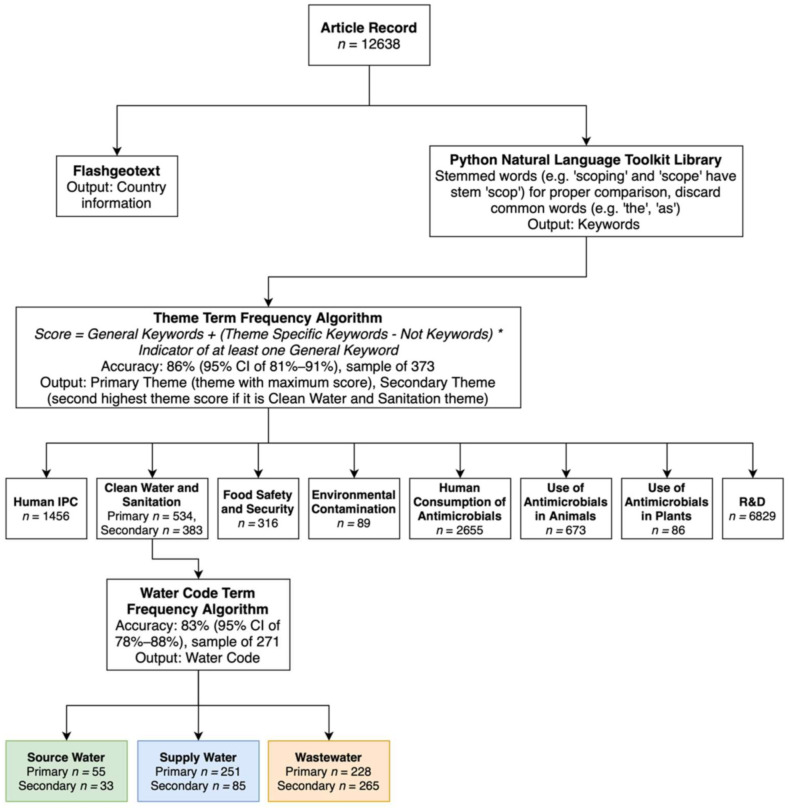
Overview of the data mining and categorization of AMR article records used in this study by geographic information, AMR themes (primary and secondary: clean water and sanitation only) and water types. The classification algorithm’s accuracy for each classification is listed along with the simple random sample used to calculate.

To analyze the current state of AMR One Health achievement, the selected article records’ titles and abstracts were mined using a frequency classification algorithm [17] to apply an AMR theme (primary and secondary) classification (Figure 2; Table S2: AMR theme classification keywords). The accuracy of this algorithm is 86% at a 95% confidence interval (Figure 2). Common and distinctive keywords were identified for each theme based on an analysis of a random sample of 10% of the total records. The theme keywords were mined using Python Natural Language Toolkit and grouped into three categories: (1) “general” keywords that could be associated with up to three themes; (2) “specific” keywords associated distinctively with one to two themes; and (3) “not” keywords to help differentiate between themes with keywords that overlap (Table S2). Root words or stems (such as “scop” in scoping and scopes) of keywords were utilized to capture terms with variable spelling. Each record’s title and abstract were reviewed against the three keyword categories using the “anded” function, and themes were determined as follows:

- Total number of “general” keywords in the title and abstract were calculated, with each “general” keyword valued at one. Themes with no general keywords were given a score of zero.
- If a record had a score of at least one, then the frequency of “anded” keywords for that article was added to the theme’s score.
- Finally, each mention of “not” keywords deducted one from the theme’s score.

Primary themes were determined based on highest scores, and secondary themes had the second highest scores (Figure 2).

Secondary theme analysis was considered only for water-related records as part of the in-depth analysis of water-associated AMR trends and One Health gaps. All water-related articles were also identified by water type with 83% accuracy at a 95% confidence interval (source, supply, or wastewater; Figure 2; Table S3: Water type classification keywords). In the One Health–One Water Stewardship Framework [10], environmental waters would be classified under the “Environmental Contamination” theme (Table 2); however, to accommodate the data mining method utilized in this review, all water-associated literature was classified under the “Clean Water and Sanitation” theme due to limitations of the classification algorithm with regards to overlap between the themes.

Geographic information, specifically country names, was extracted using Flashgeotext, and article records were categorized based on the World Bank’s income classification (high-income country (HIC); upper middle-income country (UMIC); lower middle-income country (LMIC); least developed country (LDC)) and World Bank region classification (Europe and Central Asia; South Asia; East Asia and Pacific; North America; Latin America and Caribbean; Middle East and North Africa; Sub-Saharan Africa) [18] to analyze the distribution of papers in relation to the themes and water types (Table S4: Geographic information categorization keywords). Articles were classified into multiple regions and income levels if more than one country was mentioned in the title and abstract. 

### 2.4. Global Trend Analysis of Antimicrobial Resistance Research in Relation to Themes, Region, Income, and Water Types 

Trends of countries and income levels in relation to themes and water types were analyzed using log-linear regression analysis to determine the exponential growth of article records. Specifically, the number of articles in each region, income level, and theme versus the year the articles were published were analyzed. If the number of papers was zero for a particular year, it was assumed that the logarithm of zero was −0.5. 

R squared values were also calculated to determine the quality of fit of the regression analysis. A low R squared value (<0.8) indicates that the line of best fit is a poor-quality fit, and a high R squared value (≥0.8) indicates that predictions using the line of best fit will be accurate.

## 3. Results

### 3.1. Primary Themes Prioritization

**Figure 3:**
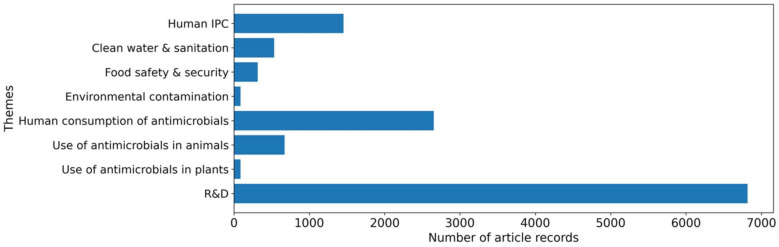
Overview of the primary theme (human infection and prevention control (IPC); research and development (R&D)) distribution among the article records (*n* = 12,638).

Within this review, the “R&D” theme has the largest proportion of articles (54%; *n* = 6829; Figure 3), followed by “Human Consumption of Antimicrobials” (21%; *n* = 2655; Figure 3) and “Human IPC” (11.5%; *n* = 1456; Figure 3). “Use of Antimicrobials in Animals” (5.3%; *n* = 673; Figure 3) and “Clean Water and Sanitation” (4.2%; *n* = 534; Figure 3) combined comprise nearly a tenth of the total records. Comparatively, “Food Safety and Security” (2.5%; *n* = 316; Figure 3), “Environmental Contamination” (0.7%; *n* = 89; Figure 3), and “Use of Antimicrobials in Plants” (0.7%; *n* = 86; Figure 3) have received limited attention in the past 30 years. Furthermore, this review found that non-food animals are under-prioritized in AMR stewardship, with only 0.3% (*n* = 41 of 12,638) of articles mentioning “wild animals” and 0.7% (*n* = 92 of 12,638) mentioning “companion animals” or “pets”. 

**Figure 4:**
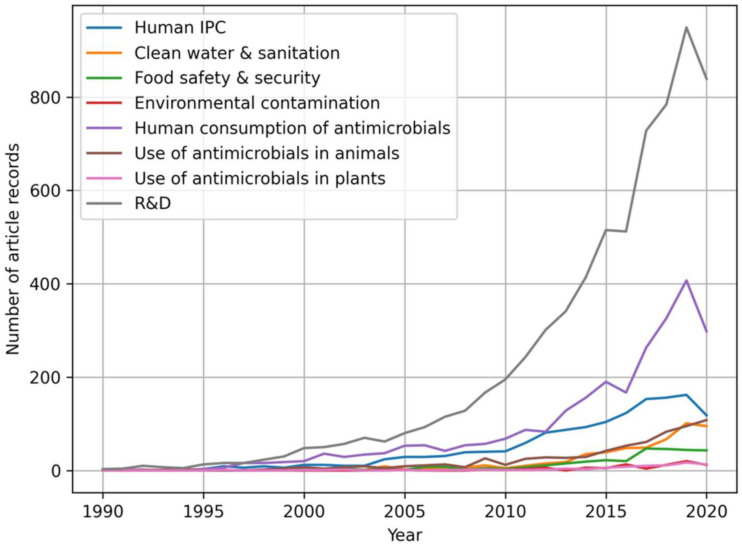
Trends of the article records from 1990–2020 in relation to AMR stewardship themes (Human Infection and Prevention Control (IPC); Research and Development (R&D)) (*n* = 12,638).

Within this review, there is an increased interest in AMR research for all themes, especially over the last decade (Figure 4). From 2010 to 2020, 84% of article records (*n* = 10,651; Figure 4) were published, compared to only 14% (*n* = 1713; Figure 4) from 2000 to 2009 and 2% (*n* = 253; Figure 4) from 1990 to 1999. This analysis also shows that “R&D” research articles account for the greatest portion of published records pertaining to AMR from 1990–2020, followed by “human consumption of antimicrobials”. However, log-linear regression analysis of the past 30 years of article records reveals that “human consumption of antimicrobials” has increased faster (m = 0.2052; Table 4) than “R&D” (m = 0.1922; Table 4), suggesting the prioritization of human-related AMR research. The “Use of Antimicrobials in Animals” has grown third fastest out of the eight themes (m = 0.1842; Table 4), followed by “Human IPC” (m = 0.1778; Table 4), while “Clean Water and Sanitation” has grown fifth fastest (m = 0.1769; Table 4). Furthermore, the “Environmental Contamination” (m = 0.1077; Table 4), “Food Safety and Security” (m = 0.1617; Table 4), and “Use of Antimicrobials in Plants” (m = 0.1055; Table 4) themes have received limited AMR research focus over the past 30 years, exhibiting the slowest exponential growth compared to the other themes (Figure 4). 

### 3.2. Global Trends in Relation to Regions and Primary Themes 

**Figure 5:**
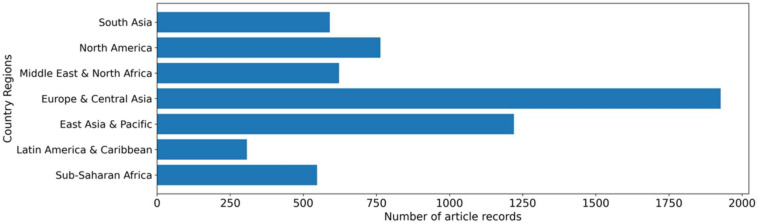
Article record distribution by World Bank regions from 1990–2020 (*n* = 12,368).

Within the reviewed articles, 43% (*n* = 5428) refer to at least one country, suggesting that AMR One Health achievements are occurring in a total of 121 countries spanning six continents (Table S5: Countries focused on AMR research and interventions from 1990–2020 by World Bank region and income classification). Most of the articles focus on the European and Central Asia region (15% of total records; *n* = 1927; Figure 5), followed by 9% (*n* = 1220; Figure 5) focusing on East Asia and the Pacific, 6% (*n* = 764; Figure 5) on North America, 5% (*n* = 622; Figure 5) on the Middle East and North Africa, 5% (*n* = 591; Figure 5) on South Asia, 4% (*n* = 547; Figure 5) on Sub-Saharan Africa, and 2% (*n* = 307; Figure 5) on Latin America and the Caribbean.

**Figure 6:**
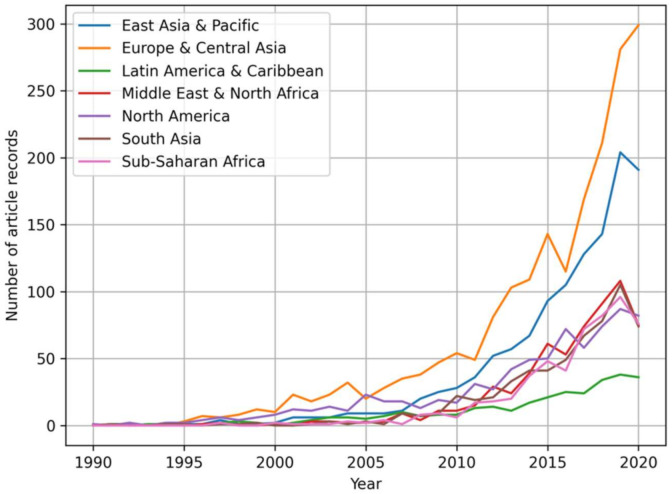
Trends of the article records from 1990–2020 by region classification (*n* = 12,368).

There is limited AMR research and knowledge dissemination globally prior to the year 2000; however, since 2010, there has been a substantial increase in AMR One Health focus (Figure 6). 

For each region, the top-three prioritized themes within this review are “r&d”, “human consumption of antimicrobials”, and “human IPC”, respectively (Table 5). The fourth to sixth prioritized themes are “use of antimicrobials in animals” followed by “clean water and sanitation” and “food safety and security”, respectively. The least prioritized themes are “use of antimicrobials in plants” and “environmental contamination” for all regions. Notably, the Middle East and North Africa region prioritizes “food safety and security” over “antimicrobial use in animals” and “clean water and sanitation”. This greater emphasis on food security reflects the Middle East and North Africa region having the world’s largest food deficit, with 25–50% of the region’s food being imported [19]. These results continue to suggest the prioritization of human and animal-focused One Health achievements in the past 30 years on a global scale, overlooking the environment, as well as plants, which are dissemination pathways to humans and animals. 

### 3.3. Global Trends in Relation to Income Distribution and Primary Themes 

**Figure 7:**
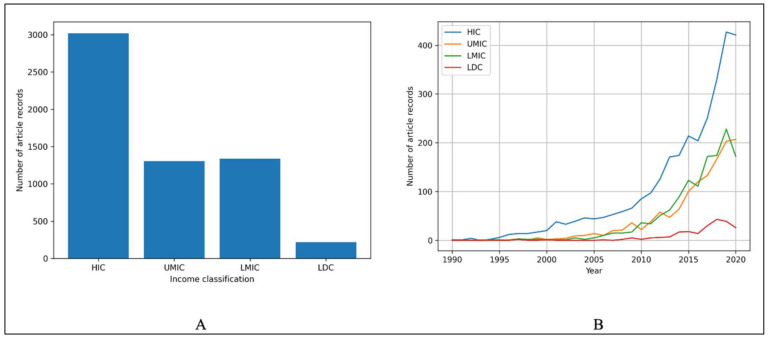
(**A**) Article record distribution by income classification (*n* = 12,368). (**B**) Trends of the article records from 1990–2020 by income classification (*n* = 12,368).

With regard to income levels, this review suggests that HICs prioritize AMR One Health by producing the most articles from 1990 to 2020, with LMIC and UMIC producing about half the number of articles and LDC producing very few in comparison (Figure 7A). Within the past 30 years, AMR research articles have been notably published in HICs beginning around the year 2000, with a drastic increase throughout the past 30 years (Figure 7B). For the UMICs and LMICs research, articles started being produced around 2005 with a more gradual increase over the 30-year period than HIC; however, a decrease in articles for LMIC started around 2017 and has continued since the 2020 cutoff within this study. LDCs research articles began publication around 2013 and have received very limited attention in the past 30 years (Figure 7B).

With respect to the log linear regression analysis, 24% (*n* = 3020; Table 6) of the total article records have an HIC focus compared to 10% (*n* = 1304; Table 6) in UMIC, 11% (*n* = 1335; Table 6) in LMIC, and 2% (*n* = 218; Table 6) in LDC. Although HICs account for the greatest proportion of total records in the past 30 years, LMICs have increased the fastest (m = 0.23; Table 6). Furthermore, UMIC One Health achievements have also increased faster (m = 0.2191; Table 6) than HICs (m = 0.1973; Table 6). Research into LDCs has grown the slowest (m = 0.1542; Table 6). These results indicate a potential financial gap in AMR One Health prioritization, limiting LMICs and LDCs.

Consistent with the overall primary theme distribution and country-specific thematic findings of this review, the most prioritized themes, in relation to income level classification, are “R&D”, “human consumption of antimicrobials”, and “human IPC”. The least prioritized themes for all income levels are “use of antimicrobials in plants” and “environmental contamination” (Table 7). HICs prioritize “use of antimicrobials in animals” (6.8%, *n* = 205; Table 7) over “clean water and sanitation” (3.7%, *n* = 114; Table 7) and “food safety and security” (2.7%, *n* = 80; Table 7). UMICs prioritize “clean water and sanitation” (6.2%, *n* = 82; Table 7) followed by an equal number of articles with the primary themes of “food safety and security” and “use of antimicrobials in animals”, which account for 4.1% (*n* = 53; Table 7) of articles each. The findings of this review suggest articles in LMICs prioritize “clean water and sanitation” (4.3%, *n* = 57; Table 7) over “use of antimicrobials in animals” (4.2%, *n* = 56; Table 7) and “food safety and security” (3.5%, *n* = 46; Table 7). LDCs articles prioritize “food safety and security” (10%, *n* = 22; Table 7) followed by an equal prioritization in the past 30 years of “use of antimicrobials in animals” (3.2%, *n* = 7; Table 7) and “clean water and sanitization” (3.2%, *n* = 7; Table 7). 

### 3.4. Global Trends in Relation to Water Type Categorization, Regions and Income Distribution 

Out of 12,638 article records, 7% (*n* = 917; Table 8) focus on water-related AMR as either a primary (4%; *n* = 534; Table 8) or secondary (3%; n = 383; Table 8) theme. Of the 917 water-related records, over half discuss One Health achievements pertaining to wastewater-related AMR risks (54%, *n* = 493; Table 8) and nearly a third pertaining to water supply concerns (37%, *n* = 336; Table 8). Only 9% (*n* = 88; Table 8) focus on source water-related AMR, indicating a potentially significant gap in interest and knowledge about AMR-associated source water risk. 

**Figure 8:**
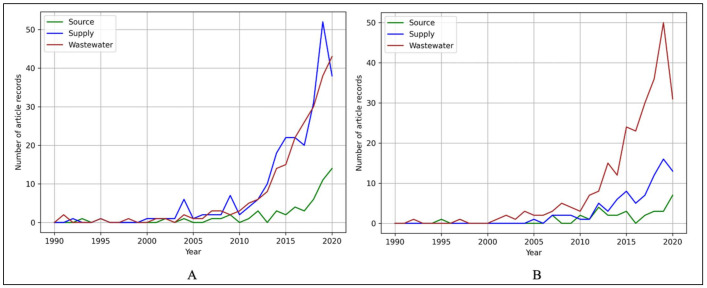
(**A**) Trends of the article records from 1990–2020 by water type classification for primary thematic focus (*n* = 534). (**B**) Trends of the article records from 1990–2020 by water type classification for secondary thematic focus (*n* = 383).

The results in this study indicate that research and knowledge about water supply and wastewater significantly increases after 2005 as a primary and secondary theme, while source water as a primary theme gradually increases after 2010 and stagnates as a secondary theme (Figure 8A,B), highlighting the source water gap in water-related AMR achievements.

With regards to regions and thematic focus for primary distribution within this study, the European and Central Asia region accounts for the greatest proportion of water-related articles (13%, *n* = 71; Table 9), followed by the East Asia and Pacific (11%, *n* = 58; Table 9) and Sub-Saharan Africa (7.6%, *n* = 41; Table 9) regions. Regions with the fewest records are Latin America and the Caribbean (3.7%, *n* = 20; Table 9) and the Middle East and North Africa (2.8%, *n* = 15; Table 9). These results indicate all regions, except for the Middle East and North Africa region, prioritize wastewater. The Middle East and North Africa region focuses primarily on water supply, which accounts for 60% of articles in this region (*n* = 9; Table 9). Source water receives the least attention in all regions, accounting for an average of 9% (Table 9) of articles. 

The secondary theme distribution shows that the Europe and Central Asia region has the most articles (13%, *n* = 50; Table 9) followed by the East Asia and Pacific region (12%, *n* = 46; Table 9). The regions with the fewest records in this study are Sub-Saharan Africa (3.9%, *n* = 15; Table 9) and the Middle East and North Africa (2.6%, *n* = 10; Table 9). These findings indicate that wastewater is prioritized across all regions. Source water again receives the least attention, with four out of seven regions having zero articles on this topic in the past 30 years within this review. 

For both primary and secondary themes in relation to income level, HICs account for the greatest proportion at 21% (*n* = 11; Table 10) and 19% (*n* = 74; Table 10), respectively. LDCs have the least records for both primary (1.3%, *n* = 7; Table 10) and secondary theme classification (0.8%, *n* = 3; Table 10). Irrespective of income level, wastewater is prioritized over water supply, and source water is the least prioritized in AMR research and interventions from 1990–2020, as indicated by these results. Furthermore, there are zero water source records from LDCs over the past 30 years. Efforts to reduce AMR disease burdens in LMICs and LDCs focus on restricting antimicrobial use, which is often coupled with calls to increase water, sanitation, and hygiene (WASH) coverage and wastewater treatment since water is recognized as a primary environmental AMR driver and dissemination pathway [11,20]. 

## 4. Discussion

### 4.1. One Health Thematic Gaps and Geographic AMR Priorities 

The United Nations Environment Programme One Health High-Level Expert Panel has improved the definition of One Health to consider domestic and wild animals, as well as plants, in accompaniment with humans, animals, and the environment [21]. Despite expanding the definition of One Health, this review found that human and animal health-focused themes are more highly prioritized, regardless of region or income level. In contrast, plant and environmental health are the least prioritized globally. Global policy and stewardship guidance similarly overprioritizes human and animal sector themes [3,5,10,16]. The global under-prioritization of environmental health in One Health AMR research and, in turn, policy and stewardship is alarming given the importance global agencies have placed on balanced multisectoral interventions to holistically address nascent AMR risks. Of particular concern is limited attention to food safety and water-related themes (“clean water and sanitation” and “environmental contamination”), especially in LMIC and LDC contexts, as LMICs and LDCs disproportionately bear the global burden of AMR risk [3]. 

The findings in this review build upon previous analyses identifying key thematic gaps in global AMR research, particularly in relation to food safety, non-food animal, and environment themes, which have been significantly overlooked to date [3,4,10,22]. Most food safety and animal research is centered on reducing human AMR risk through reduced antimicrobial use in food animal operations, despite recognized threats from plants and non-food animals. Globally, plant AMR receives the least attention, despite the alarming statistic of AMR organisms being detected on 25% of plant-based foods from all world regions [3,23]. Furthermore, this review found that non-food animals are under-prioritized in AMR research, highlighting a limitation in One Health achievements pertaining to wild and domesticated animals, which have been identified as potential human AMR exposure pathways [24,25,26]. The ongoing COVID-19 crisis serves as a critical example of the importance of addressing One Health threats of probable animal origin proactively [27,28]. 

Another important lens to consider in AMR research and integrated management is geographic priorities. Geographic priorities identified in the review interestingly reflect regional resource scarcity needs. Human health-related themes (“human IPC” and “antimicrobial consumption”) are the primary focus regardless of region; however, most regions and income levels prioritize “antimicrobial use” in food animals over “clean water and sanitation” and general “food safety and security” concerns. In relation to income level, the findings in this review highlight the continued prioritization of human and animal-related One Health achievements, thus overlooking the environment and plant food products. The greater prioritization of “clean water and sanitation” and “food safety” in UMICs, LMICs, and LDCs reflects inadequate WASH and the greater risk of AMR exposure through contaminated water and food products, unlike HICs; however, these lower income levels typically do not have the resources to implement management practices, continuing to highlight disproportioned One Health research and, in turn, management. 

To enhance efforts going forward, understanding of various AMR drivers and dissemination pathways as well as their cyclic interrelationships between the One Health sectors is needed to ensure that critical environmental aspects continue not to be overlooked in AMR stewardship [22]. A holistic and integrated approach is needed to address the global crisis of AMR in all dimensions (human, animal, plant, environment) to prevent a global decline in antimicrobial effectiveness and, in turn, human health. Environmental AMR research is limited; however, there are sufficient data to begin integrating this One Health sector through governance, surveillance, and monitoring prioritization [22]. The AMR thematic evaluation within this review provides guidance for future research interest, funding prioritization, and global guidance to prioritize the gaps in current AMR research and understanding with respect to geographic priorities. 

### 4.2. Water-Related Thematic Gaps and Geographic AMR Priorities 

Given that the environment is overlooked in AMR thematic prioritization and water resources are a primary AMR dissemination reservoir and pathway between the One Health sectors, understanding the state of water-related AMR research is a necessary step in developing effective integrated management. Not only is there a lack of emphasis on an integrated approach with thematic AMR management, this emphasis is also lacking in relation to water resources. A key aspect of integrated water resource management is the concept of One Water, which highlights the interconnected nature of water and the pollutants transported in this resource, as it fulfills the roles of source, supply, and waste. The findings within this study highlight the research focus on wastewater, which is a prominent reservoir and dissemination pathway for AMR. Wastewater has received the most attention due to the introduction and mixing of antimicrobial compounds and bacteria, resulting in AMR development and even multi-drug resistances, which are introduced to environmental source water and in turn drinking water. Regardless of this, AMR in environmental source water has been largely overlooked in the past 30 years. This is problematic from a water science and policy perspective as source water protection, which is a significant component of integrated resource management [29,30], is overlooked in global AMR understanding and stewardship as a key preventative measure. These aquatic environments are important to consider in stewardship, as a growing number of studies have identified polluted environmental waters as human, animal, and environmental AMR health threats [31,32]; however, the limited scientific understanding of this reservoir and dissemination pathway is inhibiting the development of stewardship and regulatory measures [11].

Research focus on source water protection needs to be prioritized if a holistic approach towards combating AMR is to be successful. To implement integrated water resource management including source, supply, and wastewater, reduced antimicrobial use and improved WASH and wastewater treatment is necessary. It is important to note that current statistics indicate that a quarter of the world’s population presently do not have access to safe water [33], and just over half of the world’s wastewater is treated, with large areas of Africa, Asia, Latin America, the Middle East, and Eastern Europe reporting limited treatment of, or no data on, domestic and industrial wastewater flows [34]. Additionally, treatment strategies focus on municipal and industrial effluent [20], overlooking risks from agricultural operations despite antimicrobial use in animals being triple the use in human consumption [35] and the agricultural industry using 70% of global freshwater [36], which is largely discharged as untreated runoff into the environment. Poor WASH coverage and insufficient wastewater treatment at large puts half a billion people at greater risk of AMR exposure and infection due to their reliance on potentially contaminated environmental waters to meet their daily needs [37].

Given that the One Water concept is lacking, as indicated by the off-track goals of universal WASH and wastewater treatment [38], despite acknowledgement by experts of detrimental AMR risks linked to watershed pollution [39] and water protection being recognized by FAO as the “first step” to preventing environmental AMR pollution [23], environmental water safeguards continue to be overlooked in global AMR research. There is a critical need for further prioritization and financial support from policymakers and multilateral organizations across the globe to increase research efforts and understanding of AMR risk throughout the environment, particularly water resources. This understanding is necessary to develop water protection legislation and AMR-related water quality standards that effectively surveil, prevent, and mitigate water-related AMR risks in source, supply, and wastewater [40,41], as well as equitably address human, animal, and environmental threats if an integrated One Health AMR stewardship approach is to be realized.

### 4.3. Limitations

An in-depth synthesis of research within each theme was not possible within the scope of this study, which aimed to highlight the current focus of AMR research globally. The large sample (*n* = 12,638) also limited an in-depth synthesis of the available research outside of the water-related content, necessitating a data mining approach. Data mining presents limitations that include overfitting and potential overlap between theme classification terms due to equal weighting of terms or bias. The latter was accounted for and addressed using the scoring system outlined in the method section, and accuracy for the theme and water type classification algorithm was calculated and reflected in the findings of the review to represent potential for error or bias. Regression models or neural networks can be used for more unbiased weighting estimations; however, this could not be applied in this study as it was beyond the scope and due to time and resource constraints.

## 5. Conclusions

The findings in this review indicate that the current state of AMR prioritization is centered on human and animal health, disregarding the One Health purview implemented to combat this global health crisis. There is a significant focus on research and development, which is contributing to understanding AMR, developing new antimicrobial compounds, and AMR surveillance; however, these advancements typically pertain to human and animal AMR research. Food safety, antimicrobial use in plants, environmental contamination, and clean water and sanitation are extensively overlooked in AMR research, which inhibits a holistic and comprehensive One Health understanding of AMR risk and stewardship needs.

Furthermore, research has overlooked water resources, especially environmental waters, as global reservoirs and dissemination pathways. Within the limited water-related AMR research, wastewater is prioritized over supply water, and environmental source water receives the least attention, indicating siloed management not only in the One Health sectors but also within water resources management. This finding indicates that the environmental focus in One Health achievements is potentially limited, preventing an integrated approach. A multi-sector One Health approach cannot be achieved without sufficient understanding of all reservoirs and dissemination pathways—human, animal, plant, environmental, and water resources—through scientific research.

It is recommended that AMR research pertaining to food safety and security, use of antimicrobials in plants, domestic and wild animals as reservoirs and dissemination pathways, as well as the environment, including air, soil, and water resources, be effectively integrated into developing research, stewardship, and integrated management to ensure critical environmental aspects are prioritized. In particular, greater research prioritization of preventative environmental and source water contamination is needed, as source water is likely a critical dissemination pathway of environmental AMR. Further research can innovatively apply data mining methods to facilitate the review of AMR research in which key gaps in knowledge and stewardship can be readily identified to support the implementation of integrated and holistic stewardship in accordance with a One Health approach.

## Figures and Tables

**Table 1:** Antimicrobial resistance research and interventions—rapid review inclusion and exclusion criteria for manual and secondary screening process.

	Inclusion Criteria	Exclusion Criteria
**Population**	Global, especially LMIC Human, animal and/or environmental studies with evident link to human AMR risks (e.g., food products)	Studies with no evident link to human AMR risks
**Intervention**	AMR methods, risk surveillance and risk reduction activities (scientific or programmatic) from industries/sectors associated with AMR source, spread and transmission AMR must feature in results’ objective and/or outcomes	Biocides and heavy metals as origins of AMR (i.e., not environmental pollution intermediary)
**Outcome**	AMR knowledge contribution (e.g., evaluative study, method), control, surveillance, or risk reduction/mitigation of public health risks	Inclusion of AMR should not be solely as consequence in rationale
**Date range**	January 1990-September 2020, from when AMR was framed as a “shared global problem” [15]	Earlier than 1990
**Publication type**	Title and abstract for peer-reviewed publications (including randomized control trials, case controls, systematic reviews, review article (on a topic/subject), articles-in-press, qualitative case studies, policy briefs, etc.), conference proceedings (research papers and summative reports), data papers, corrected articles Language: English	Books, chapters, book reviews, special issue overviews, journal supplements, commentaries, editorials, letters (to editors), review protocols, magazine articles, theses, errata, notes, retracted or original articles if correction is available, conference proceeding abstracts/overviews, webinars, meeting minutes, presentations

**Table 2:** Antimicrobial resistance stewardship themes and focus areas.

Themes	Focus Areas
Human Infection Prevention and Control (IPC)	Clinical development of AMR and preventative measures to limit the spread of human infection in healthcare settings through effective control practices, including handwashing and personal protective equipment.
Clean water and sanitation	Clean water, sanitation, and hygiene (WASH) access; wastewater treatment; and environmental waters.
Food safety and security	Food safety and security in the food chain can expose humans to AMR organisms through food handling or consumption.
Environmental contamination	Soil and air can facilitate AMR exposure through three primary mechanisms: as a transmission vector; introduction of selective pressures (e.g., heavy metals or antibiotics) for the development of AMR and highly resistant strains (such as superbugs); and as a reservoir of antimicrobial resistant genes.
Human consumption of antimicrobials	Prescription, dispensing, misuse, improper dosage, improper diagnosis, and consumption of antimicrobials within humans, which is increasing the ineffectiveness of available antimicrobials.
Use of antimicrobials in animals	Antimicrobials used in food animal production for disease prevention, growth promotion, or prophylactic group treatment.
Use of antimicrobials in plants	Use of antimicrobials in food plant production. Plant products can be infected with resistant pathogens from the application of mass crop sprays or fertilizers.
Antimicrobial agents, drugs, and tools research and development (R&D)	Development of novel or alternative products (e.g., antimicrobials, vaccines), understanding resistance mechanisms within all One Health sectors (e.g., genes, peptides, biofilms), and the development and implementation of new methods (e.g., detection, surveillance).

**Table 3:** Defined water types and examples in relation to antimicrobial resistance.

Water Type	Definition	Examples
Source	Includes water resources within the environment (environmental or ambient waters) that may or may not be impacted by anthropogenic influence, such as pathogens and other pollutants (nonpoint and point source).	Surface water (lakes, rivers, streams), marine water (oceans, seas, estuaries, brackish water), groundwater, and karst groundwater resources
Supply	Includes treated or untreated water supply intended for human use and consumption, as well as industrial use.	Treated municipal supply, well water, and self-supplied water (i.e., glacial melt, rainwater harvesting, direct use of surface water)
Wastewater	Includes polluted or used water being reintroduced to environmental waters. This may be water that has encountered fecal waste, pathogens, heavy metals, and pharmaceutical byproducts.	Stormwater runoff, agricultural runoff/drainage, and industrial, pharmaceutical, municipal, and hospital wastewater

**Table 4:** Logarithmic linear regression slope and R squared values for each theme in relation to the trend analysis of the article records from 1990–2020.

Theme	Slope (m)	R Squared Value	Color Ranking by Exponential Growth
Human IPC	0.1778	0.9197	Fastest (0.18+)
Clean Water and Sanitation	0.1769	0.8484	
Food Safety and Security	0.1617	0.8385	
Environmental Contamination	0.1077	0.6519	Medium (0.15–0.18)
Human Consumption of Antimicrobials	0.2052	0.8991	
Use of Antimicrobials in Animals	0.1842	0.9258	Slowest (0–0.15)
Use of Antimicrobials in Plants	0.1055	0.7237	
R&D	0.1922	0.9794	

**Table 5:** AMR theme distribution of article records and ranking by regions (*n* = 12,638).

Theme:	R&D	Human Consumption of Antimicrobials	Human IPC	Use of Antimicrobials in Animals	Clean Water and Sanitation	Food Safety & Security	Use of Antimicrobials in Plants	Environmental Contamination
**Region**								
Europe and Central Asia	855	604	186	147	71	39	16	9
South Asia	296	148	75	20	29	15	7	1
East Asia and Pacific	634	278	134	61	58	43	8	4
North America	329	215	101	41	29	36	8	5
Latin America and Caribbean	171	50	35	12	20	14	3	2
Middle East and North Africa	358	130	130	17	15	21	0	0
Sub-Saharan Africa	228	124	86	27	41	35	4	2
**Color ranking of themes by number of articles**								
1st		2nd	3rd	4th	5th	6th	7th	8th

**Table 6:** Logarithmic linear regression slope and R squared values for each income level in relation to the trend analysis of the article records from 1990–2020.

Income Level	Slope (m)	R Squared Value	Color Ranking by Exponential Size
HIC	0.1973	0.9141	Fastest (0.2+)
UMIC	0.2191	0.9657	Medium (0.16–0.2)
LMIC	0.2300	0.9238	Slowest (0–0.16)
LDC	0.1542	0.7668	

**Table 7:** AMR theme distribution of article records and ranking by income level (*n* = 12,638).

Theme:	R&D	Human Consumption of Antimicrobials	Human IPC	Clean Water and Sanitation	Food Safety and Security	Environmental Contamination	Use of Antimicrobials in Animals	Use of Antimicrobials in Plants
**Region**								
**HIC**	1319	938	325	114	80	0	205	24
**UMIC**	704	253	145	82	53	6	53	8
**LMIC**	720	273	165	57	46	0	56	8
**LDC**	91	51	36	7	22	1	7	3
**Color ranking of themes by number of articles**								
1st		2nd	3rd	4th	5th	6th	7th	8th

**Table 8:** Water type distribution within the water-related AMR article records by primary or secondary categorization of the clean water and sanitation theme (*n* = 12,638).

Clean Water and Sanitation Theme Ranking	Water Type: Source	Water Type: Supply	Water Type: Wastewater	Total Article Records by Theme Ranking
Primary	55	251	228	534
Secondary	33	85	265	383
**Total Article Records by Water Type**	**88**	**336**	**493**	**917**

**Table 9:** Water type distribution ranking of water-related article records by primary and secondary theme categorization of the clean water and sanitation theme in relation to regions (*n* = 917).

	Region:	Europe and Central Asia	South Asia	East Asia and Pacific	North America	Latin America and Caribbean	Middle East and North Africa	Sub-Saharan Africa
	**Water** **Category**							
**Primary Theme** **Categorization**	Source	4	4	6	3	1	2	2
	Supply	32	12	20	11	9	9	11
	Wastewater	35	13	32	15	10	4	28
	**Total Articles**	**71**	**29**	**58**	**29**	**20**	**15**	**41**
**Secondary Theme** **Categorization**	Source	0	1	2	0	0	0	1
	Supply	13	2	7	5	5	2	3
	Wastewater	37	26	37	14	11	8	11
	**Total Articles**	**50**	**29**	**46**	**19**	**16**	**10**	**15**
	**Color ranking of water categories by number of articles**							
	1st			2nd		3rd		

**Table 10:** Water type distribution ranking of water-related article records by primary and secondary theme categorization of the clean water and sanitation theme in relation to income level (*n* = 917).

	Income Level:	HIC	UMIC	LMIC	LDC
	**Water Category**				
**Primary Theme Categorization**	Source	9	8	6	0
	Supply	51	29	19	2
	Wastewater	54	45	32	5
	**Total Articles**	**114**	**82**	**57**	**7**
**Secondary Theme** **Categorization**	Source	0	2	2	0
	Supply	21	10	4	0
	Wastewater	53	48	37	3
	**Total Articles**	**74**	**60**	**43**	**3**
	**Color ranking of water categories by number of articles**				
	1st		2nd	3rd	

## Data Availability

The data presented in this study are available in Figure 1, Figure 2, Figure 3, Figure 4, Figure 5, Figure 6, Figure 7 and Figure 8, Table 1, Table 2, Table 3, Table 4, Table 5, Table 6, Table 7, Table 8, Table 9 and Table 10, and Table S5.

## Supplementary Materials

The following supporting information can be downloaded at: https://www.mdpi.com/article/10.3390/ijerph19116549/s1, Table S1: Database search keywords and search strings; Table S2: AMR theme classification keywords; Table S3: Water type classification keywords; Table S4: Geographic information categorization keywords; Table S5: Countries focused on AMR research and interventions from 1990–2020 by World Bank region and income classification.
