# Supplementary material for: A Rapid Review of Environmental Health Gaps in Antimicrobial Resistance and Water-Related Research from 1990–2020
Source: Int J Environ Res Public Health. 2022 May 27;19(11):6549. doi: 10.3390/ijerph19116549 (PMC9180282; doi:10.3390/ijerph19116549)
Supplement: Supplementary file 1 [file ijerph-19-06549-s001.zip › ijerph-1704195-supplementary.pdf]

Table S1: Database Search Keywords and Search Strings

- Keywords/search terms per concepts: AND across, OR down.
- Keywords based on literature from the pharmaceutical, medical, agricultural and water industries.
- Limitations: (1) search of Title, Abstract and subject headings/keywords only; and (2) publication period from 1990-2020.

**Antimicrobial Resistance (AMR)** *AMR synonyms*	**AND**	**AMR-associated science** *(1) topics (e.g. risk factors, gene transfer); (2) sector-specific terms, actions or methods employed (e.g. genom* analysis, treat*), including analytical frameworks.*	**AND**	**AMR-associated interventions** *(1) actions addressing resistance; (2) programs (e.g. antimicrobial stewardship); and (3) Impact. Can possibly include (4) policy outputs. (Decided to exclude.)*
(antibiot* or "anti-biot*" or "anti-microbial*" or antimicrobial* or drug or pandrug or "pan-drug" or "multi-drug") W/2 (resistan*)		**Mechanisms of organism resistance:**<br><br>(mutat* or "natural selection" or emerg*) W/3 (spread or transmi*)		**Addressing resistance (I.e. AMR function):**<br><br>(remov* or target* or treat* or reduc* or prevent* or mitiga* or control or manage*) W/2 (antibiot* or "anti-biot*" or antimicrob* or "anti-microb*")
		**Human risk factors**:<br><br>(diagnos* or prescrib* or overuse or dispens* or misuse or consum* or inject*) W/2 (antibiot* or "anti-biot*" or antimicrob* or "anti-microb*")		(remov* or target* or treat* or reduc* or prevent* or mitiga* or control or manage*) W/3 resist*
		**Economic risk factors:**<br><br>(manufact* or distribut* or agriculture or aquaculture or agricultur* or processing or transport*) W/3 (antibiot* or "anti-biot*" or antimicrob* or "anti-microb*")		**Programs/Interventions:**<br><br>program* or surveillance or "antimicrobial stewardship" or "anti-microbial stewardship" or "antibiotic stewardship" or "anti-biotic stewardship" or "behavio* chang*" or "research and development"
		**Environmental sources/reservoirs of resistance:**<br><br>(environment* or soil or water or feed or fertiliser* or fertilizer* or manure or waste or sewage) W/2 (contamina* or pollut*)		**Societal impact / academic contribution:**<br><br>"public health" or "environmental sanitation" or "environmental health" or "planetary health" or "one health" or "implementation science"
		**Evaluation of resistance or defense:**		**Policy outputs:**

		(“susceptibility test*” or “sensitivity test” or peptide or screen* or detect* or inactivat*) W/2 (antibiot* or “anti-biot*” or antimicrob* or “anti-microb*”)		protocol or regulat* or standard* or guideline* or strateg*

Table S2: AMR Theme Classification Keywords

	General keywords (1 point each)	And keywords (1 point each)	Not keywords (-1 point each mention)
Human infection prevention and control	infection, prevention, control, personal, protective, equipment, PPE, disinfection, sterilization, sanitization, cleaning, washing, mask, hospital-acquired, nosocomial, HIV, AIDs, viral, antiviral, prophylaxis	human, children, infant, adult, student, doctor, nurse, dentist, pharmacist, patient, resident, hospital, ICU, long-term, clinic, clinician, health-care, healthcare, health, facility, surface, contact, hygiene	use, usage, consumption, consume, prescribe, overuse, misuse, access, dosage, practice, prescription, immunization, vaccine, slaughter, butcher, butchery, packaging, retail, street, grocery, food, foodborne, food-borne, pork, beef, dairy, mutton, meat, seafood, shops, store, market, food-producing, slaughterhouse
Human consumption of antimicrobials	use, usage, consumption, consume, prescribe, overuse, misuse, treatment, dosage, access, practice, prescription, stewardship, immunization, vaccine, behaviour, behavior, decision	human, children, infant, adult, student, doctor, nurse, dentist, pharmacist, patient, resident, hospital, ICU, long-term, clinic, clinician, health-care, healthcare, health, facility, physician	infection, prevention, personal, protective, equipment, PPE, disinfection, sterilization, sanitization, cleaning, washing, mask, hospital-acquired, nosocomial, HIV, AIDs, viral, antiviral, prophylaxis, surface, contact, hygiene, probiotic, farm, farmland, aquaculture, aquacultural, husbandry, veterinary, zoonotic, zoonoses, zoonosis, CAFO, concentrated, feeding, operation, confined, manure, excreta, excrement, animal, livestock, poultry, bird, rabbit, pig, cow, bovine, ovine, lamb, fish, chicken, piglet, shrimp, oyster, pet, companion, canine, feline, cats, dogs, feces, cattle, wildlife, boar, broiler, mammals, horses, pesticide, herbicide, fungicide, biocide, fertilizer, agriculture, agricultural, pond, tank, nursery, horticulture, harvest, plant, fruit, vegetable, crop, leaf, leaves, legume, root, produce, seaweed, kelp, algae
Clean water & sanitation	karst, river, lake, stream, marine, groundwater, influent, drinking, potable, water, wastewater, waste, sewage, effluent, landfill, aquifer	contamination, manure, excrement, excreta, excrement, leach, leachate, pollution, run-off, runoff, feces, treatment, removal, filtration, chlorination, ultraviolet, irradiation, oxidation, transmission, clean, sanitation, supply, source, surface, reservoir	wildlife, eDNA, environment, ecology, ecosystem, soil

Environmental contamination	wildlife, remediation, removal, eDNA, environment, ecology, ecosystem, soil	contamination, manure, excrement, excreta, excrement, leach, leachate, pollution, run-off, runoff, sewage, feces, transmission, reservoir	karst, river, lake, stream, marine, influent, drinking, potable, water, wastewater, effluent, landfill, aquifer
Food safety & security	slaughter, butcher, butchery, packaging, retail, street, grocery, food, foodborne, food-borne, pork, beef, dairy, mutton, meat, seafood, shops, store, market, food-producing, slaughterhouse	contamination, surface, hygiene, animal, chickens, farm, consumption, supply, safety, security, production, chain, handling, processing, import, export	
Use of antimicrobials in animals	use, usage, consumption, consume, prescribe, overuse, misuse, treatment, dosage, access, practice, prescription, stewardship, immunization, vaccine, behaviour, behavior	probiotic, farm, farmland, aquaculture, aquacultural, husbandry, veterinary, zoonotic, zoonoses, zoonosis, CAFO, concentrated, feeding, operation, confined, manure, excreta, excrement, animal, livestock, poultry, bird, rabbit, pig, cow, bovine, ovine, lamb, fish, chicken, piglet, shrimp, oyster, pet, companion, canine, feline, cats, dogs, feces, cattle, wildlife, wild, boar, broiler, mammals, horses	human, children, infant, adult, student, doctor, nurse, dentist, pharmacist, patient, resident, hospital, ICU, long-term, clinic, clinician, health-care, healthcare, health, facility, physician, pesticide, herbicide, fungicide, biocide, fertilizer, manure, agriculture, agricultural, pond, tank, nursery, horticulture, harvest, plant, fruit, vegetable, crop, leaf, leaves, legume, root, produce, seaweed, kelp, algae
Use of antimicrobials in plants	use, usage, consumption, consume, prescribe, overuse, misuse, treatment, dosage, access, practice, prescription, stewardship, immunization, vaccine, behaviour, behavior	pesticide, herbicide, fungicide, biocide, fertilizer, manure, farm, agriculture, agricultural, aquaculture, aquacultural, pond, tank, nursery, horticulture, harvest, plant, fruit, vegetable, crop, leaf, leaves, legume, root, produce, seaweed, kelp, algae	human, children, infant, adult, student, doctor, nurse, dentist, pharmacist, patient, resident, hospital, ICU, long-term, clinic, clinician, health-care, healthcare, health, facility, physician, probiotic, husbandry, veterinary, zoonotic, zoonoses, zoonosis, CAFO, concentrated, feeding, operation, confined, excreta, excrement, animal, livestock, poultry, bird, rabbit, pig, cow, bovine, ovine, lamb, fish, chicken, piglet, shrimp, oyster, pet, companion, canine, feline, cats, dogs, feces, cattle, wildlife, boar, broiler, mammals, horses
Antimicrobial agents, drugs, and tools research and development	research, development, R&D, genesis, evolution, sequence, nanotechnology-based, trend, model, deterministic, review, scope, study, detect, diagnostic, mutation, alternative, agent, susceptibility, activity, activation, activate, activator, deactivate, deactivation, deactivator, inactivation, inactivate, mechanism, gene, genome, genomic, genotype, nucleotype, peptide, plasmid, serotype, serovar, pathogen, carbapenem, biofilm, efflux, pump	antibiotic, antimicrobial, antibacterial, anti-biotic, anti-microbial, anti-bacterial, multidrug, multi-drug, drug, vaccine, therapeutic, novel, method	use, usage, consumption, consume, prescribe, overuse, misuse, dosage, access, practice, prescription, stewardship, behaviour, behavior, infection, prevention, control, personal, protective, equipment, PPE, disinfection, sterilization, sanitization, cleaning, washing, mask, hospital-acquired, nosocomial, HIV, AIDs, viral, antiviral, prophylaxis, surface, contact, hygiene

Table S3: Water Type Classification Keywords

Water Code	Subcategory	Keywords
Source	Surface Water	surface, river, lake, stream, tributary, creek
	Marine Water	sea, ocean, saline, estuary, brackish, salt
	Groundwater	aquifer, groundwater, confined, unconfined, granular, alluvial
	Karst Groundwater	Karst, aquifer, groundwater, limestone, carbonate, dissolution-type, landscape
Supply	Municipal	household, drinking, human, treatment, distribution, regulated
	Well	well, private, self-regulated
	Self-supplied	glacier, fog, rainwater, cistern, river
Wastewater	Agricultural	Irrigation, run-off, animal, waste, manure, feed, by-product, pesticides, herbicides, insecticides, nutrient
	Industrial	industrial, waste, heavy, metals, high, dissolved, solids, organic, compounds, toxic, energy, production
	Municipal	wastewater, sewage, wastewater, treatment, plant, household, human, waste, primary, secondary, tertiary
	Pharmaceutical	drug, development, antibiotic, byproduct
	Stormwater	surface, runoff, urban, industrial, agriculture, nonpoint, source, precipitation, rainfall, drainage, injection
	Hospital	clinical, waste, antibiotics, viruses

Table S4: Geographic Information Categorization Keywords

Regions	Keywords
Sub-Saharan Africa	Benin, Botswana, Burkina Faso, Cameroon, Cape Verde, Central African Republic, Chad, Comoros, Democratic Republic of Congo, Eswatini, Ethiopia, Gabon, Ghana, Guinea-Bissau, Ivory Coast, Kenya, Lesotho, Madagascar, Malawi, Republic of Congo, Rwanda, South Africa, Sudan, Tanzania, Zambia, Zimbabwe
Latin America & Caribbean	Argentina, Bolivia, Brazil, Chile, Colombia, Costa Rica, Cuba, Jamaica, Mexico, Peru, Puerto Rico, Suriname, Trinidad and Tobago
East Asia & Pacific	Australia, Brunei, Cambodia, China, East Timor, Indonesia, Japan, Malaysia, Myanmar, New Zealand, Philippines, Singapore, South Korea, Taiwan, Thailand, Vanuatu, Vietnam
Europe & Central Asia	Albania, Austria, Azerbaijan, Belarus, Belgium, Bosnia and Herzegovina, Bulgaria, Croatia, Czech Republic, Denmark, Estonia, Finland, France, Georgia, Germany, Greece, Hungary, Iceland, Ireland, Italy, Kazakhstan, Kosovo, Kyrgyzstan, Latvia, Lithuania, Luxembourg, Montenegro, Netherlands, Norway, Poland, Portugal, Romania, Russia, Serbia, Slovakia, Slovenia, Spain, Sweden, Switzerland, Turkey, Ukraine, United Kingdom
North America	Canada, United States
South Asia	Afghanistan, Bangladesh, Bhutan, India, Nepal, Pakistan, Sri Lanka

Middle East & North Africa	Algeria, Bahrain, Egypt, Iran, Iraq, Israel, Jordan, Lebanon, Morocco, Oman, Palestine, Saudi Arabia, Tunisia, United Arab Emirates
**Income level classification**	**Keywords**
High Income Country (HIC)	Australia, Austria, Bahrain, Belgium, Brunei, Canada, Chile, Croatia, Czech Republic, Denmark, Estonia, Finland, France, Germany, Greece, Hungary, Iceland, Ireland, Israel, Italy, Japan, Latvia, Lithuania, Luxembourg, Netherlands, New Zealand, Norway, Oman, Poland, Portugal, Puerto Rico, Saudi Arabia, Singapore, Slovakia, Slovenia, South Korea, Spain, Sweden, Switzerland, Taiwan, Trinidad and Tobago, United Arab Emirates, United Kingdom, United States
Upper Middle-Income Country (UMIC)	Albania, Argentina, Azerbaijan, Belarus, Bosnia and Herzegovina, Botswana, Brazil, Bulgaria, China, Colombia, Costa Rica, Cuba, Gabon, Georgia, Iraq, Jamaica, Jordan, Kazakhstan, Kosovo, Lebanon, Malaysia, Mexico, Montenegro, Peru, Romania, Russia, Serbia, South Africa, Suriname, Thailand, Turkey
Lower Middle-Income Country (LMIC)	Algeria, Bangladesh, Benin, Bhutan, Bolivia, Cambodia, Cameroon, Cape Verde, Comoros, East Timor, Egypt, Eswatini, Ghana, India, Indonesia, Iran, Ivory Coast, Kenya, Kyrgyzstan, Lesotho, Morocco, Myanmar, Nepal, Pakistan, Palestine, Philippines, Republic of Congo, Sri Lanka, Tanzania, Tunisia, Ukraine, Vanuatu, Vietnam, Zambia, Zimbabwe
Least Developed Country (LDC)	Afghanistan, Burkina Faso, Central African Republic, Chad, Democratic Republic of Congo, Ethiopia, Guinea-Bissau, Madagascar, Malawi, Rwanda, Sudan

Table S5: Countries focused on AMR research and interventions from 1990-2020 by World Bank region and income classification

Region	Country	Income Level
East Asia and Pacific	Australia	High Income
	Brunei	High Income
	Cambodia	Lower Middle-Income
	China	Upper Middle-Income
	East Timor	Lower Middle-Income
	Indonesia	Lower Middle-Income
	Japan	High Income
	Malaysia	Upper Middle-Income
	Myanmar	Lower Middle-Income
	New Zealand	High Income
	Philippines	Lower Middle-Income
	Singapore	High Income
	South Korea	High Income
	Taiwan	High Income
	Thailand	Upper Middle-Income
	Vanuatu	Lower Middle Income
	Vietnam	Lower Middle Income

**Europe and Central Asia**	Albania	Upper Middle-Income
	Austria	High Income
	Azerbaijan	Upper Middle-Income
	Belarus	Upper Middle-Income
	Belgium	High Income
	Bosnia and Herz.	Upper Middle-Income
	Bulgaria	Upper Middle-Income
	Croatia	High Income
	Czech Republic	High Income
	Denmark	High Income
	Estonia	High Income
	Finland	High Income
	France	High Income
	Georgia	Upper Middle-Income
	Germany	High Income
	Greece	High Income
	Hungary	High Income
	Iceland	High Income
	Ireland	High Income
	Italy	High Income
	Kazakhstan	Upper Middle-Income
	Kosovo	Upper Middle-Income
	Kyrgyzstan	Lower Middle-Income
	Latvia	High Income
	Lithuania	High Income
	Luxembourg	High Income
	Montenegro	Upper Middle-Income
	Netherlands	High Income
	Norway	High Income
	Poland	High Income
	Portugal	High Income
	Romania	Upper Middle-Income
	Russia	Upper Middle-Income
	Saudi Arabia	Upper Middle-Income
	Slovakia	High Income
	Slovenia	High Income
	Spain	High Income
	Sweden	High Income
	Switzerland	High Income
	Turkey	Upper Middle-Income
	Ukraine	Lower Middle Income
	United Kingdom	High Income
**Latin America and Caribbean**	Argentina	Upper Middle-Income
	Bolivia	Lower Middle-Income
	Brazil	Upper Middle-Income
	Chile	High Income
	Colombia	Upper Middle-Income
	Costa Rica	Upper Middle-Income
	Cuba	Upper Middle-Income
	Jamaica	Upper Middle-Income
	Mexico	Upper Middle-Income
	Peru	Upper Middle-Income
	Puerto Rico	High Income
	Suriname	Upper Middle-Income
	Trinidad and Tobago	High Income

Middle East and North Africa	Algeria	Lower Middle-Income
	Bahrain	High Income
	Egypt	Lower Middle-Income
	Iran	Lower Middle-Income
	Iraq	Upper Middle-Income
	Israel	High Income
	Jordan	Upper Middle-Income
	Lebanon	Upper Middle-Income
	Morocco	Lower Middle-Income
	Oman	High Income
	Palestine	Lower Middle-Income
	S. Sudan	High Income
	Tunisia	Lower Middle Income
	United Arab Emirates	High Income
North America	Canada	High Income
	United States of America	High Income
South Asia	Afghanistan	Least Developed
	Bangladesh	Lower Middle-Income
	Bhutan	Lower Middle-Income
	India	Lower Middle-Income
	Nepal	Lower Middle-Income
	Pakistan	Lower Middle-Income
	Sri Lanka	Lower Middle-Income
Sub-Saharan Africa	Benin	Lower Middle-Income
	Botswana	Upper Middle-Income
	Burkina Faso	Least Developed
	Cameroon	Lower Middle-Income
	Cape Verde	Lower Middle-Income
	Central African Rep.	Least Developed
	Chad	Least Developed
	Comoros	Lower Middle-Income
	Dem. Rep. Congo	Least Developed
	eSwatini	Lower Middle-Income
	Ethiopia	Least Developed
	Gabon	Upper Middle-Income
	Ghana	Lower Middle-Income
	Guinea-Bissau	Least Developed
	Ivory Coast	Lower Middle-Income
	Kenya	Lower Middle-Income
	Lesotho	Lower Middle-Income
	Madagascar	Least Developed
	Malawi	Least Developed
	Republic of Congo	Lower Middle-Income
	Rwanda	Least Developed
	South Africa	Upper Middle-Income
	Sudan	Least Developed
	Tanzania	Lower Middle-Income
	Zambia	Lower Middle Income
	Zimbabwe	Lower Middle Income
